# The impact of tocilizumab treatment on the severity of inflammation and survival rates in sepsis is significantly influence by the timing of administration

**DOI:** 10.1007/s10787-025-01649-6

**Published:** 2025-02-15

**Authors:** Taha Tavaci, Zekai Halici, Elif Cadirci, Mustafa Ozkaraca, Kamber Kasali

**Affiliations:** 1 Faculty of Medicine, Sakarya University, Sakarya, Türkiye; 2 Faculty of Medicine, Atatürk University, Erzurum, Türkiye; 3 Faculty of Veterinary Medicine, Cumhuriyet University, Sivas, Türkiye

**Keywords:** Sepsis, Tocilizumab, IL-6, Inflammation, Immunity, Treatment timing

## Abstract

**Background:**

Sepsis is a life-threatening organ dysfunction resulting from a dysregulated host response to infection. Due to the high mortality rates and treatment costs associated with sepsis, research is focusing on innovative treatment strategies to replace one dimensional approaches. Recent studies are being conducted on the use of immunotherapeutics in sepsis and the impact of treatment timing. This study aimed to elucidate the significance of treatment timing in sepsis immunotherapy with Tocilizumab (TCZ) and the implications of differences in treatment timing.

**Methods:**

LPS-induced sepsis model was established in rats to assess the changes in interleukin-6 (IL-6) over a 24-h sepsis period and its correlation with lung and kidney injury. The impact of TCZ treatments at various time points was evaluated by molecular and histopathological methods. The effect of TCZ treatment timing on survival was analyzed using Kaplan–Meier survival analysis.

**Results:**

IL-6 reached peak concentrations in the early stages of sepsis, whereas lung damage peaked subsequent to the IL-6 peak, and kidney damage manifested considerably later. The early treatment group, receiving intervention one hour post-sepsis induction, exhibited the most favorable molecular and histopathological outcomes. Conversely, the group receiving the latest treatment, at sixteen hours post-sepsis induction, demonstrated the poorest results. Survival analysis indicated that the group treated at the tenth hour exhibited the highest survival rate.

**Conclusion:**

Variations in the timing of sepsis treatment with TCZ yield significantly different molecular outcomes, histopathological results, and survival rates. A thorough investigation of the timing of immunotherapeutic applications in sepsis treatment will enhance the efficiency of sepsis treatments.

## Introduction

Sepsis is a life-threatening organ dysfunction resulting from heterogeneous host immune responses to infection. The disease remains a significant global health priority and the subject of numerous contemporary studies; however, its pathogenesis, involving the immune system, is not yet fully understood. The elevated mortality rates and treatment costs associated with sepsis patients, who exhibit divergent immune responses ranging from hyperinflammation to immunoparalysis, necessitate a departure from conventional, one-dimensional treatments in favor of effective, innovative therapeutic approaches (Tavaci and Akgun 2022).

The pleiotropic cytokine interleukin-6 (IL-6) is a clinically diagnostic biomarker that precisely reflects inflammatory burden. It increases significantly in sepsis, correlating with disease severity, and is associated with organ dysfunction and sepsis-related mortality (Huang et al. 2020; Weidhase et al. 2019; Yu et al. 2022). The excessive release of IL-6 induces the inflammatory IL-6 signaling pathway, leading to organ and tissue damage and systemic complications. However, a drug has been developed that suppresses this inflammatory pathway. Tocilizumab (TCZ), an immunosuppressive drug and humanized anti-IL-6 receptor antibody of the IgG1 subclass, inhibits the binding of IL-6 to its receptors, diminishing the proinflammatory activity of cytokines by competing for the soluble and membrane-bound forms of the human IL-6 receptor (Venkiteshwaran 2009). In clinical trials and off-label applications, TCZ has demonstrated significant efficacy as a treatment option for diseases characterized by excessive IL-6 secretion (Chen et al. 2016).

TCZ has been extensively utilized to mitigate the significant inflammatory burden during the COVID-19 pandemic (Klopfenstein et al. 2021). According to certain meta-analysis results examining the use of TCZ in COVID-19 treatment, there was no significant difference in mortality rate, need for mechanical ventilation, and ICU admission rate between COVID-19 patients who received TCZ treatment and without TCZ treatment; however, other meta-analyses indicated a reduction in these parameters for patients receiving TCZ treatment (Lan et al. 2020; Group 2021). Although the use of TCZ is similar in dose and regimen, it has been argued that the variation in results may be attributable to differences in the timing of drug administration (Abidi et al. 2022). The timing of immunosuppressive therapy is crucial in sepsis treatment owing to the disease’s immunopathogenesis, comparable to COVID-19 (Zafer et al. 2021). Given the opposing immune responses in sepsis, it is plausible that two patients with simultaneous sepsis induction could exhibit divergent immune reactions concurrently. Therefore, the timing of drug administration in sepsis treatment should reflect the immune state (e.g., hypo or hyperinflammation) rather than a numerical expression. By incorporating this distinction into immunotherapy, personalized treatment for sepsis can be achieved. Although this may deviate from standardized approaches, appropriate treatment timing in sepsis should be based on suppressing excessive hyperinflammatory responses or preventing the patient from entering immunosuppression. When examining the paradigm of immune responses in sepsis, it is evident that the prevailing notion is that the host immune response is characterized by an initial overwhelming inflammatory phase that evolves into a longer immunosuppressive phase within a few days (Hotchkiss et al. 2013). Therefore, all anti-cytokine immunosuppressive drugs—but TCZ in particular—must be administered within the “golden hour” window.

The determination of whether a patient with sepsis is in the hyperinflammation phase or experiencing immunoparalysis can be achieved through the use of specific biomarkers (Trzeciak et al. 2020). IL-6 serves as a valuable biomarker of both diagnostic and prognostic significance for sepsis and septic shock (Song et al. 2019c).

Moreover, elevated IL-6 levels associated with mortality in sepsis patients and IL-6’s efficacy in predicting sepsis mortality have demonstrated that it plays a direct role in human sepsis (Song et al. 2019b; Vivas et al. 2021; Naffaa et al. 2013). A recent Mendelian randomization study indicated that sepsis treatment with IL-6 receptor antagonists may be advantageous (Hamilton et al. 2023). However, no study in the literature has optimized variables other than the timing of treatment and evaluated sepsis treatment with TCZ application at different times. In this study, we posited that the immune status of a sepsis patient could be ascertained by monitoring temporal variations in serum levels of the biomarker, and that treatment efficacy would be enhanced through optimal timing determined by analyzing the correlation between these temporal biomarker variations and organ injury. Our hypothesis was experimentally tested by administering TCZ treatments at various time points in rats with lipopolysaccharide (LPS)-induced sepsis, using IL-6 as a biomarker.

## Materials and methods

### Materials

LPS was obtained from Sigma-Aldrich (L2880, Lipopolysaccharides from Escherichia coli O55:B5). TCZ was purchased form Roche (Actemra 200 mg/10 mL IV Concentrate Vial). Rat IL-6 Enzyme-Linked Immuno Sorbent Assay (ELISA) kit was obtained from Elabscience (Cat.No.:E-EL-R0015). Rat interleukin-1β (IL-1β) ELISA kits (Cat No.:SL0402Ra), rat IL-6 ELISA kits (Cat No.:SL0411Ra), rat tumor necrosis factor α (TNF-α) ELISA kits (Cat No.:SL0722Ra), and rat nuclear factor kappa-light-chain-enhancer of activated B cells p65 (NFκβ-p65) ELISA kits (Cat No.: SL0538Ra) were purchased from SunLong Biotech. Large Volume Detection System anti-Polyvalent, horseradish peroxidase (HRP) was obtained from Thermofischer (catalog no: TP-125-HL). Mayer’s hematoxylin was purchased from Sigma-Aldrich (MHS32). Janus kinase 2 (JAK2) monoclonal antibody (E-AB-22272), phospho Janus kinase 2 (pJAK2) polyclonal antibody (E-AB-68172), and signal transducer and activator of transcription 3 (STAT3) monoclonal antibody (E-AB-22119) were purchased from Elabscience. Phospho signal transducer and activator of transcription 3 (pSTAT3) monoclonal antibody (sc-8059), and suppressor of cytokine signaling 3 (SOCS-3) monoclonal antibody (sc-518020) were obtained from Santa Cruz.

### Animals

All animal experiments were conducted in accordance with EU and Turkish legal and ethical regulations following governmental approval of the experimental protocol. The study was approved and authorized by the Atatürk University Local Animal Care Commission on June 30, 2022 (decision no. 145; meeting no. 2022/7). A total of 292 male rats (Wistar albino, 7–8 weeks old and weighing between 220 and 250 g) were used in this study. The animals were obtained from Atatürk University Medical and Experimental Application and Research Center.

### Experimental design

The experiments comprised three principal studies. The initial study included 13 groups, each group containing 6 rats. LPS at a dose of 10 mg/kg was administered intraperitoneally into the right lower quadrant of the rats using a 21-gauge needle to establish an LPS-induced sepsis model. Following the sepsis induction, each group was euthanized at 2-h intervals from hour 0 to hour 24, and blood, lung, and kidney tissues were collected. In the second study, based on correlation tests between IL-6 levels from the first study and organ injury levels in the lung and kidney, the 1st, 6th, 8th, 10th, and 16th hours were selected as time points for TCZ treatment following the sepsis induction with LPS. This second study examined the effects of TCZ treatment at different times, comparing the healthy, sepsis, and 1st-, 6th-, 8th-, 10th-, and 16th-hour groups. Following to the sepsis induction with LPS, TCZ was administered intraperitoneally at the specified times in a 1 cc isotonic sodium chloride solution at a dose of 2 mg/kg per rat. After 24 h following to the sepsis induction with LPS, all groups were anesthetized with 100 mg/kg ketamine and 10 mg/kg xylazine, and lung and kidney tissue samples were obtained. The third study entailed a survival analysis post-sepsis induction, involving groups receiving TCZ treatment at the 1st, 6th, 8th, 10th, and 16th hours, alongside the healthy, healthy + TCZ, and sepsis groups. The rats were monitored for 72 h following sepsis induction.

### Molecular analysis

In the first study, IL-6 levels in blood tissue serum samples were measured following the kit protocol, and the concentration was calculated as pg/ml. In the subsequent study, the lung and kidney tissue samples were pulverized using a molecular homogenizer in liquid nitrogen. The pulverized tissues were homogenized three times for 90 s with a phosphate-buffered saline homogenate solution in a transparent capped plastic tube, followed by centrifugation at a maximum speed for 10 min. IL-1β, IL-6, TNF-α, and NFκβ-p65 levels from the obtained supernatants were measured using a spectrophotometry device via the ELISA method, and the concentration was calculated as pg/mg. The ELISA method was carried out according to the protocol outlined in the manual and included in the kit.

### Histopathological and immunohistochemical analysis

The lung and kidney tissue samples were preserved in a neutral formalin (10%) solution. The tissues underwent routine processing with alcohol and xylene and were embedded in paraffin blocks. Sections of 4 μm were placed on slides and stained with hematoxylin–eosin dye for histopathological evaluation. Preparations for the lung and kidney tissue samples from the second study were performed as previously described (Keskin et al. 2022). Subsequently, the tissues were washed with phosphate-buffered saline solution and incubated overnight at + 4ºC with JAK2 monoclonal antibody, pJAK2 polyclonal antibody, STAT3 monoclonal antibody, pSTAT3 monoclonal antibody, and SOCS-3 monoclonal antibody at a dilution ratio of 1/150. According to the manufacturer’s instructions, large volume detection system anti-polyvalent, HRP was used as a secondary antibody. DAB (3,3′-Diaminobenzidine) was used as a chromogen. Following counterstaining with Mayer’s hematoxylin, the sections were mounted with entellan and examined under a light microscope. The examination focused on the immunopositivity detected in the lungs and kidneys.

### Statistical method

SPSS 20 and the OriginLab statistical analysis program were utilized for all data analyses. The data were presented as standard deviations in the statistical analyses of the ELISA results. The normality distribution of continuous variables was examined using the Shapiro–Wilk test and the Kolmogorov–Smirnov test. To compare continuous variables with three or more independent groups, the ANOVA test was employed when the normal distribution condition was met, and the Kruskal–Wallis test was used when it was not. Post hoc tests following ANOVA were performed using the Tukey test when variances were homogeneous, and Tamhane’s T2 test when variances were not homogeneous. The Kruskal–Wallis one-way ANOVA (k samples) test was employed for post hoc tests following the Kruskal–Wallis test. Correlation tests were conducted using Pearson correlation if the normal distribution condition was met when comparing two quantitative variables and the Spearman correlation test if it was not. For 24-h analysis, hierarchical agglomerative clustering and heat map dendrogram techniques were used, with z scores and correlation values provided. Survival analysis was performed using the Kaplan–Meier method. In the statistical analysis of histopathological findings and immunohistochemical staining results, differences between groups were determined using the Kruskal–Wallis test, a nonparametric test, and the group responsible for the difference was identified using the Mann–Whitney U test. The statistical significance level was set at p < 0.05 in all tests.

## Results

### The changes in 24-h serum IL-6, lung injury, and kidney injury levels in sepsis and their relationship

**Fig. 1:**
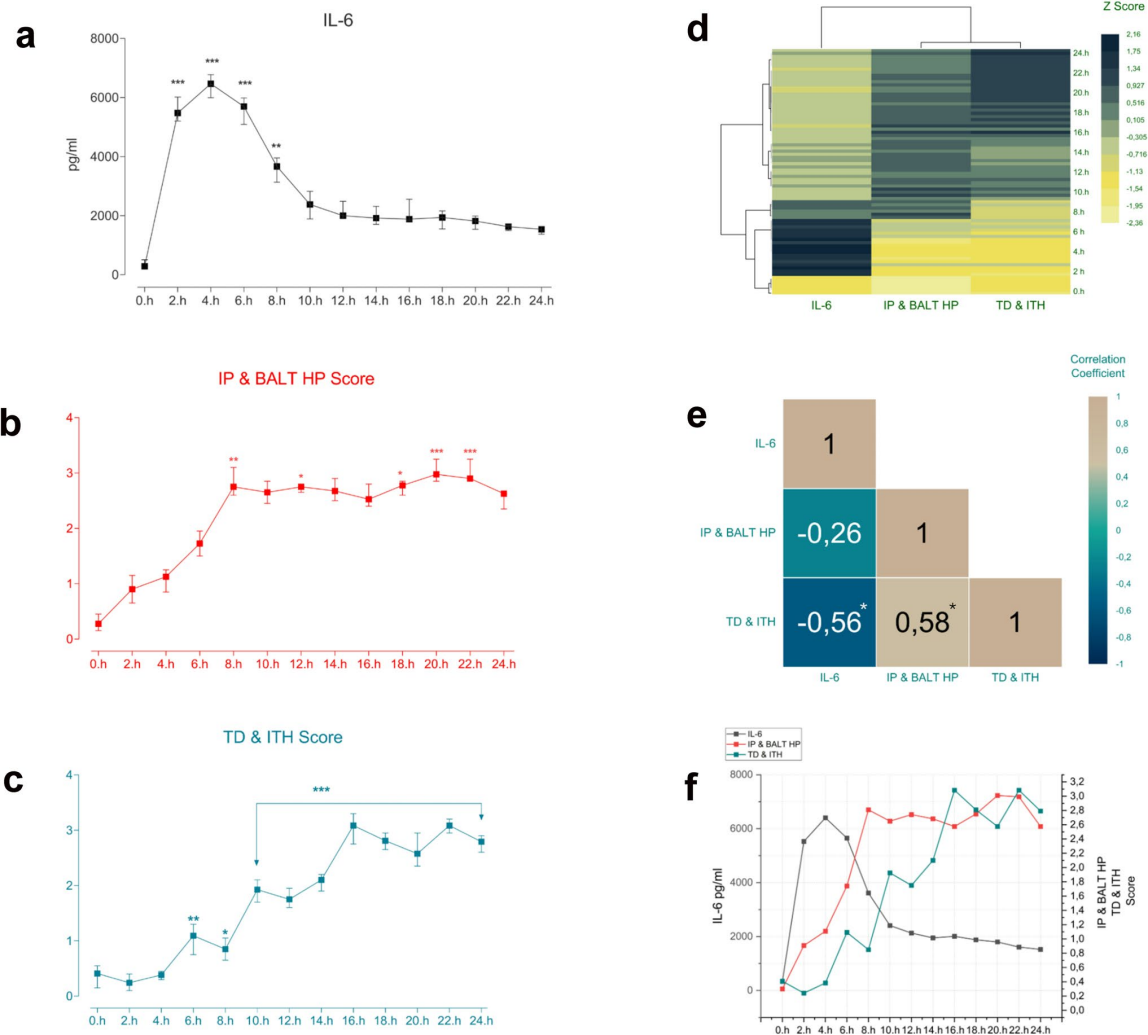
Alterations in IL-6, pulmonary injury, and renal injury during sepsis and their interrelationships. **a** Measurement of serum IL-6 levels over a 24-h period during sepsis. **P < 0.01 and ***P < 0.001 vs. hour 0. **b** Examination of IP&BALT HP (Interstitial Pneumonia & Bronchus-Associated Lymphoid Tissue Hyperplasia) scores during 24-h sepsis. *P < 0.05, **P < 0.01 and ***P < 0.001 vs. hour 0. **c** Examination of TD&ITH (Tubular Degeneration & Intertubular Hemorrhage) scores during 24-h sepsis. *P < 0.05, **P < 0.01 and ***P < 0.001 vs. hour 0. **d** Examination of changes in IL-6, lung injury, and kidney injury over time during 24-h sepsis using the hierarchical clustering method. **e** Examination of the relationship between IL-6, lung injury, and kidney injury over time during 24-h sepsis using the heatmap plot. **f** Graphical representation of alterations in IL-6 levels, IP&BALT HP score, and TD&ITH score over a 24-h sepsis period

The serum profile of the IL-6 in sepsis was monitored at narrow intervals over 24 h. Serum IL-6 levels increased significantly at 2nd hour compared to hour 0 following sepsis induction; IL-6 levels peaked at 4th hour and were higher at 6th hour compared to 2nd hour but lower than at 4th hour. IL-6 levels decreased significantly at 8th and 10th hours compared to 6th hour, and this decrease continued gradually and more slowly from 12th hour until the end of the 24th hour (Fig. 1a). The course of lung injury in sepsis was monitored with interstitial pneumonia (IP) and bronchus-associated lymphoid tissue hyperplasia (BALT HP) scores. Following sepsis induction, it was observed that IP&BALT HP scores increased slightly as of the 2nd hour, and this increase continued regularly at the 4th, 6th, and 8th hours. IP&BALT HP scores, which peaked at the 8th hour, remained at the same level until the end of the 24th h (Fig. 1b). The course of kidney injury in sepsis was monitored with tubular degeneration (TD) and intertubular hemorrhage (ITH) scores. It was observed that the TD&ITH scores did not change within the first 4 h following sepsis induction but increased from the 6th to the 16th hour. The increase in TD&ITH scores peaked at the 16th hour and remained at approximately the same level until the end of the 24th hour (Fig. 1c). The data were presented using hierarchical clustering to better observe the behavior of IL-6, lung injury, and kidney injury during the sepsis process and better understand their changes. During the sepsis process, IL-6 was observed at high levels between the 2nd and 6th hours, and a decrease in IL-6 levels began to occur from the 8th hour. While high IL-6 values were observed in the early hours of sepsis, IL-6 levels decreased in the late period of sepsis. Lung injury, unlike IL-6, was at low levels in the early period of sepsis, peaked at the 8th hour, and continued at the same level until the end of the 24th hour. Kidney injury, on the other hand, exhibited low levels in the early hours of sepsis, increased to a moderate level of injury by the 10th hour, and peaked at the 16th hour, remaining at the same level until the end of the 24th hour (Fig. 1d). A heatmap plot was created to examine the relationship between IL-6, lung injury, and kidney injury during sepsis. A moderate negative correlation between lung injury and serum IL-6 levels was observed. There is a statistically significant negative correlation between kidney injury and IL-6 levels. Regarding the relationship between lung and kidney injury, a statistically significant positive correlation was found (Fig. 1e).

### Timing differences in TCZ treatment yield different results on sepsis-induced inflammatory burden in the lungs

**Fig. 2:**
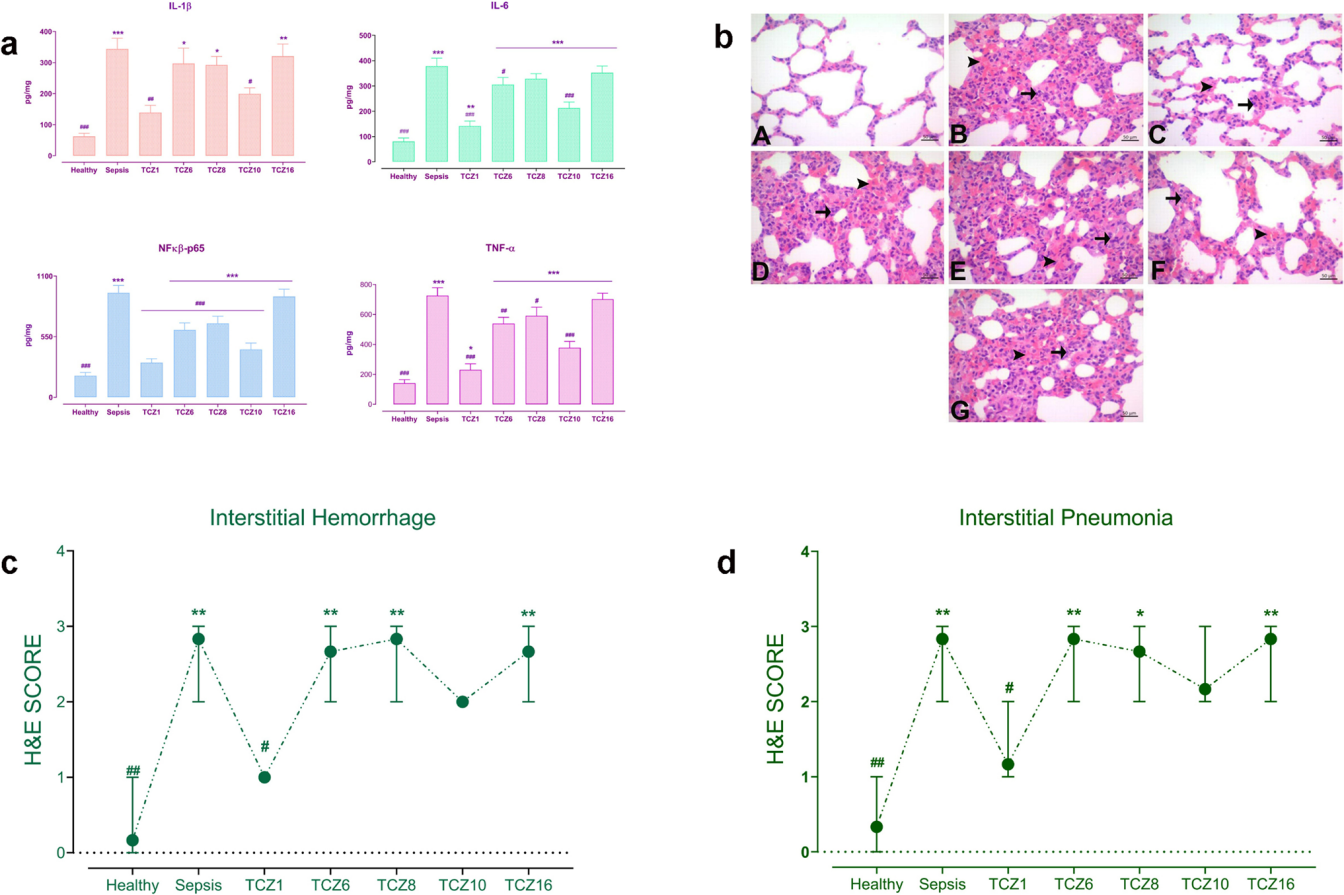
Molecular and histopathological implications of TCZ treatment on lung tissue. **a** Measurement of IL-1β, IL-6, NFκβ-p65, and TNF-α concentrations in lung tissue. *P < 0.05, **P < 0.01 and ***P < 0.001 vs. healthy group. #P < 0.05, ##P < 0.01 and ###P < 0.001 vs. sepsis group. **b** Lung histopathology of TCZ treatment groups. A- healthy group: normal histological appearance. B- sepsis group: severe interstitial pneumonia and hemorrhage. C- TCZ-1 group: mild interstitial pneumonia and hemorrhage. D- TCZ-6 group: severe interstitial pneumonia and hemorrhage. E- TCZ-8 group: severe interstitial pneumonia and hemorrhage. F- TCZ-10 group: moderate interstitial pneumonia and hemorrhage. G- TCZ-16 group: severe interstitial pneumonia and hemorrhage. (Interstitial pneumonia: → , hemorrhage: ►) **c** Graph depicting interstitial hemorrhage scores. **P < 0.01 vs. healthy group. #P < 0.05 and ##P < 0.01 vs. sepsis group. **d** Graph depicting interstitial pneumonia scores. *P < 0.05 and **P < 0.01 vs. healthy group. #P < 0.05 and ##P < 0.01 vs. sepsis group

It was observed that timing differences in TCZ treatment yielded varying results on sepsis-induced lung injury (Fig. 2). IL-1β levels in the lung tissue were significantly elevated in the sepsis group compared to the healthy group. While no significant increase was observed in the TCZ1 and TCZ10 groups compared to the healthy group, a statistically significant increase in IL-1β levels was noted in the TCZ6, TCZ8, and TCZ16 groups. IL-6 levels were significantly elevated in the sepsis group and all TCZ-treated groups compared to the healthy group, with a lower increase in the TCZ1 group than in the sepsis and other TCZ treatment groups. NFκβ-p65 levels were significantly elevated in all TCZ-treated groups, except the sepsis and TCZ1 groups, compared to the healthy group. Significant differences were noted between all groups except the TCZ16 and sepsis groups. TNF-α levels were significantly elevated in the sepsis and all TCZ-treated groups compared to the healthy group, with a lower increase in the TCZ1 group than in the sepsis and TCZ treatment groups. Significant differences were observed between all groups except for the TCZ16 and sepsis groups (Fig. 2a). Interstitial pneumonia and interstitial hemorrhage levels, examined using the hematoxylin–eosin staining method, were significantly elevated in the sepsis group compared to the healthy group. No significant increase was detected in the TCZ1 and TCZ10 groups compared to the healthy group, whereas a statistically significant increase was observed in the TCZ6, TCZ8, and TCZ16 groups (Fig. 2b, c, d).

### Timing differences in TCZ treatment yield different results on sepsis-induced inflammatory burden in the kidneys

**Fig. 3:**
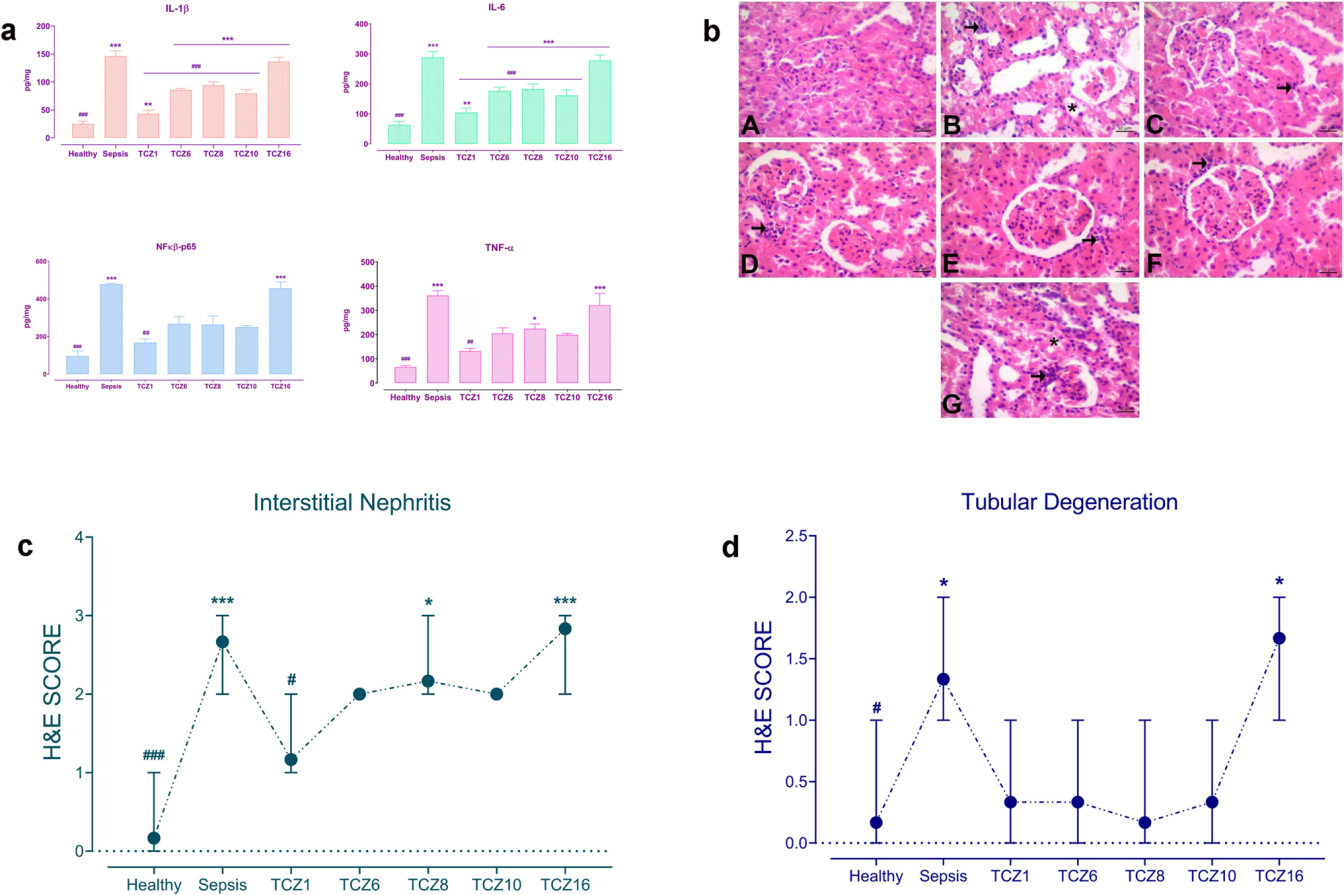
Molecular and histopathological implications of TCZ treatment on kidney tissue. **a** Measurement of IL-1β, IL-6, NFκβ-p65, and TNF-α concentrations in kidney tissue. *P < 0.05, **P < 0.01 and ***P < 0.001 vs. healthy group. #P < 0.05, ##P < 0.01 and ###P < 0.001 vs. sepsis group. **b** Kidney histopathology of TCZ treatment groups. A- Healthy group, normal histological appearance. B- Sepsis group, severe interstitial nephritis and moderate tubular degeneration. C- TCZ-1 group, mild interstitial nephritis. D- TCZ-6 group, E- TCZ-8 and F- TCZ-10 groups, moderate interstitial nephritis. G- TCZ-16 group, severe interstitial nephritis and moderate tubular degeneration. (Interstitial nephritis: → , tubular degeneration: ►). **c** Graph depicting interstitial nephritis scores. *P < 0.05 and ***P < 0.001 vs. healthy group. #P < 0.05 and ###P < 0.001 vs. sepsis group. **d** Graph depicting tubular degeneration scores. *P < 0.05 vs. healthy group. #P < 0.05 vs. sepsis group

The impact of varied timing in TCZ treatment on sepsis-induced kidney injury was evaluated by measuring proinflammatory cytokine levels in the kidney tissue and histopathological analysis (Fig. 3). IL-1β levels in the kidney tissue were significantly elevated in the sepsis group and all TCZ-treated groups compared to the healthy group. The elevation in IL-1β levels was lower in the TCZ1 group compared to the sepsis group and the other TCZ treatment groups. IL-6 levels were significantly increased in the sepsis group and all TCZ-treated groups relative to the healthy group. The increase in IL-6 levels was lower in the TCZ1 group compared to the sepsis group and other TCZ treatment groups. NFκβ-p65 levels were elevated in the sepsis group compared to the healthy group; however, there was no significant elevation in NFκβ-p65 levels in the TCZ1, TCZ6, TCZ8, and TCZ10 groups relative to the healthy group, while a statistically significant increase was observed in the TCZ16 group compared to the healthy group. Examination of TNF-α levels revealed a substantial increase in the sepsis group compared to the healthy group. There was no significant increase in TNF-α levels in the TCZ1, TCZ6, and TCZ10 groups relative to the healthy group. However, there was a statistically significant increase in TNF-α levels in the TCZ8 and TCZ16 groups compared to the healthy group (Fig. 3a). Interstitial nephritis and tubular degeneration were assessed using hematoxylin and eosin staining in the kidney tissues (Fig. 3b,c,d). Interstitial nephritis levels were significantly elevated in the sepsis group compared to the healthy group. There was no significant increase in interstitial nephritis levels in the TCZ1, TCZ6, and TCZ10 groups relative to the healthy group, but a statistically significant elevation was noted in the TCZ8 and TCZ16 groups compared to the healthy group (Fig. 3c). Tubular degeneration levels were significantly higher in the sepsis group than in the healthy group. There was no significant increase in tubular degeneration levels in the TCZ1, TCZ6, TCZ8, and TCZ10 groups relative to the healthy group, but a statistically significant increase was observed in the TCZ16 group compared to the healthy group (Fig. 3d).

### The JAK2/STAT3/SOCS3 pathway is modulated in the lungs by differences in TCZ administration timing in sepsis

**Fig. 4:**
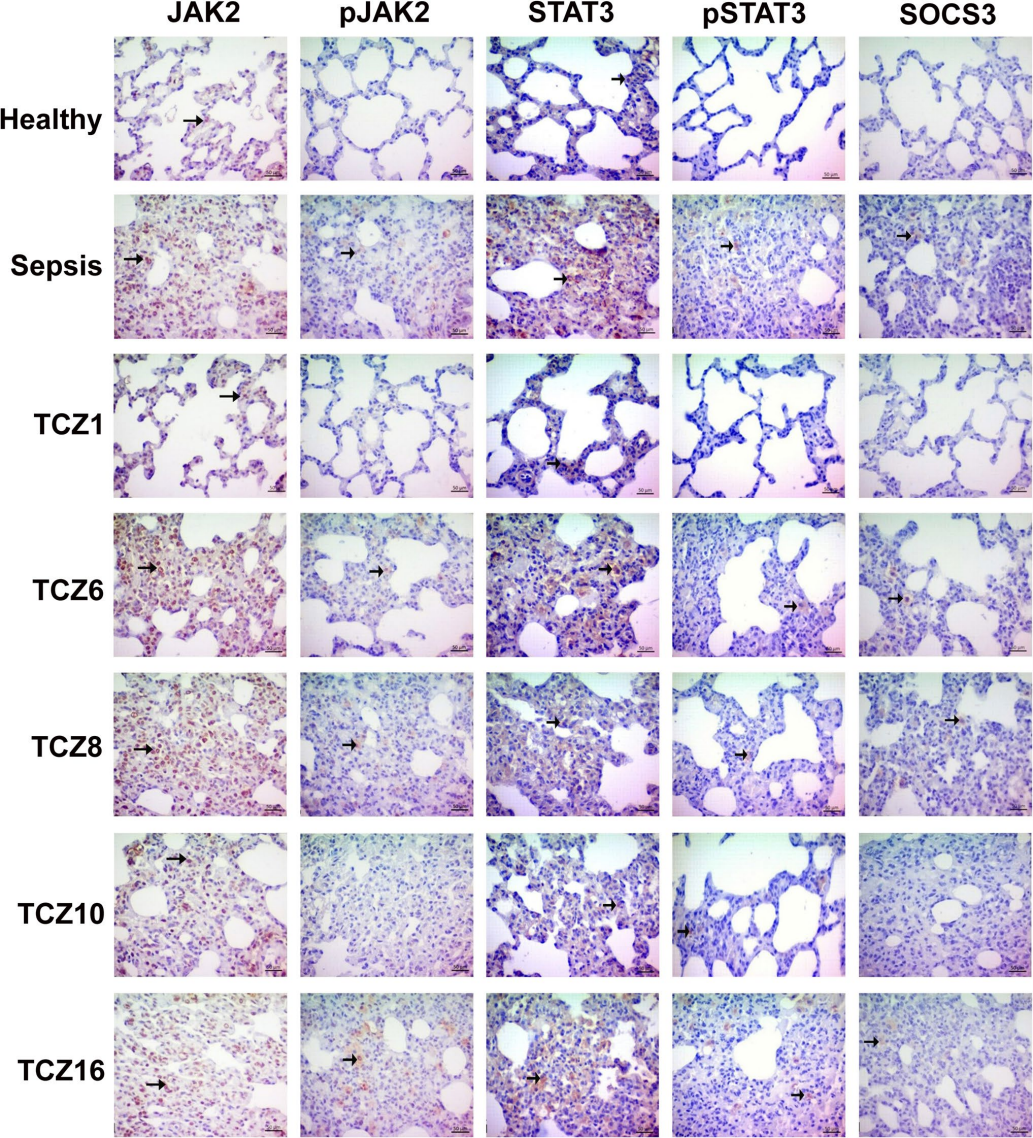
Modulation of the JAK2/STAT3/SOCS3 pathway in lung tissue by TCZ treatment. ( →) denotes immunohistochemical staining

JAK2 protein expression in the lung tissue exhibited a significant increase in the sepsis group compared to the healthy group, whereas the TCZ1, TCZ10, and TCZ16 groups did not differ significantly from the healthy group. Examination of pJAK2 levels revealed no significant difference between the sepsis and healthy groups; however, a statistically significant difference was identified between the healthy group and the TCZ16 group. STAT3 protein expression in the lung tissue was significantly elevated in the sepsis group relative to the healthy group. While the TCZ1 and TCZ10 groups did not show a significant difference compared to the healthy group, a statistically significant difference was observed between these groups and the sepsis group. Analysis of pSTAT3 levels indicated a significant difference in the sepsis group compared to the healthy group. The TCZ1, TCZ6, TCZ8, and TCZ10 groups showed no significant variance from the healthy group, whereas the TCZ16 group was statistically significantly different than the healthy group. Examination of SOCS3 protein expression indicated a significant increase in the sepsis group compared to the healthy group (Fig. 4) (Table 1).

**Table 1:** The JAK2/STAT3/SOCS3 expression in lung tissues of TCZ treatment groups was measured by immunohistochemistry

Groups	JAK2	pJAK2	STAT3	pSTAT3	SOCS3
Healthy	0,83 ± 0,40^aA^	0,16 ± 0,40^aA^	0,83 ± 0,40^aB^	0,00 ± 0,00^aA^	0,00 ± 0,00^aA^
Sepsis	3,00 ± 0,00^bA^	1,33 ± 0,51^bB^	3,00 ± 0,00^bA^	1,83 ± 0,40^bC^	1,00 ± 0,00^bB^
TCZ1	1,16 ± 0,40^aA^	0,00 ± 0,00^aB^	0,83 ± 0,40^aA^	0,00 ± 0,00^aB^	0,16 ± 0,40^aB^
TCZ6	3,00 ± 0,00^cA^	1,00 ± 0,00^bB^	2,16 ± 0,40^cC^	0,83 ± 0,40^cB^	0,83 ± 0,40^bB^
TCZ8	3,00 ± 0,00^cA^	1,16 ± 0,40^bB^	2,00 ± 0,00^cC^	0,83 ± 0,40^cB^	0,83 ± 0,40^bB^
TCZ10	1,83 ± 0,40^dA^	0,16 ± 0,40^aB^	1,16 ± 0,40^aC^	0,83 ± 0,40^cC^	0,16 ± 0,40^aB^
TCZ16	2,83 ± 0,40^bA^	2,16 ± 0,40^cB^	2,16 ± 0,40^cB^	1,83 ± 0,40^bB^	0,66 ± 0,51^bC^

a, b, c, d denote differences between groups in the same column (p < 0.05). A, B, C denote differences between groups in the same row (p < 0.05)

### The JAK2/STAT3/SOCS3 pathway is modulated in the kidneys by differences in TCZ administration timing in sepsis

**Fig. 5:**
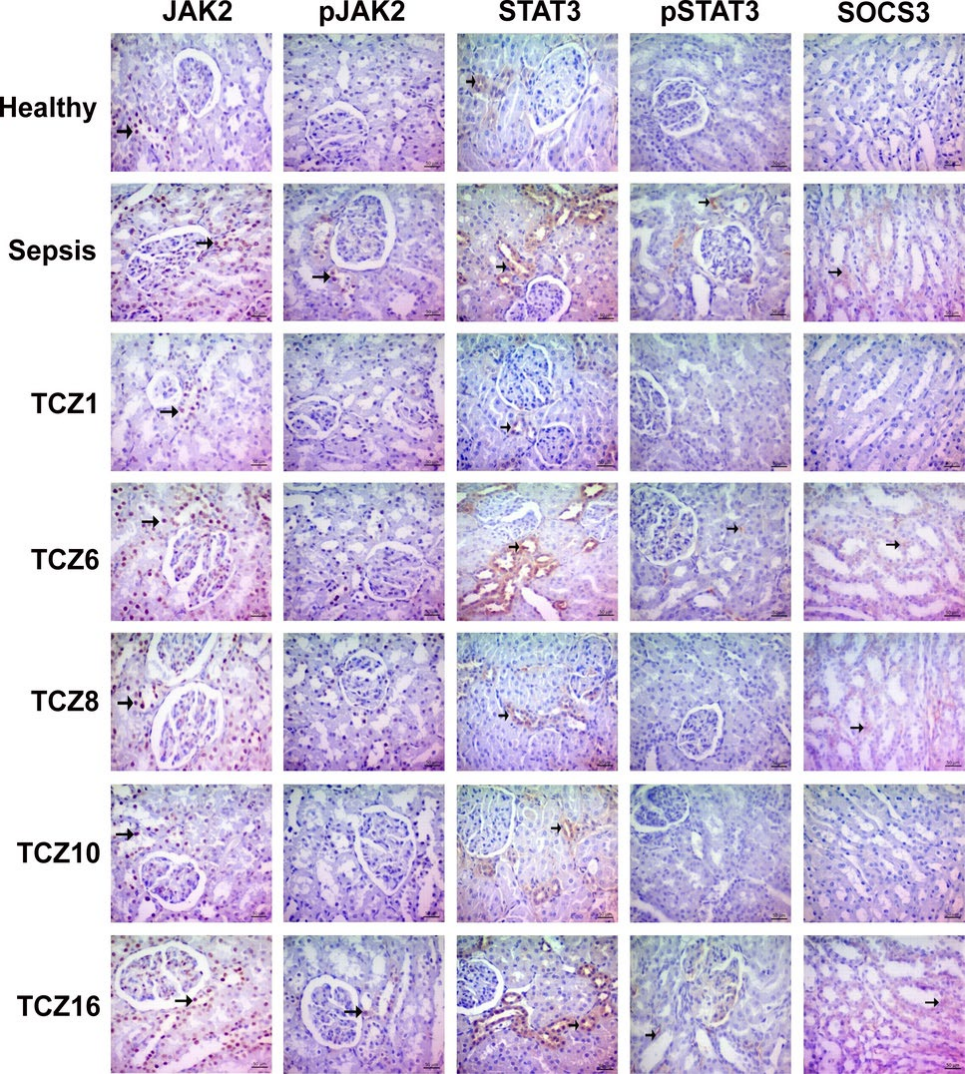
Modulation of the JAK2/STAT3/SOCS3 pathway in kidney tissue by TCZ treatment. ( →) denotes immunohistochemical staining

In the kidney tissue, JAK2 protein expression exhibited a significant increase in the sepsis group compared to the healthy group. There was no significant increase in the TCZ1 and TCZ10 groups compared to the healthy group. Examination of pJAK2 levels revealed no significant differences between the groups. STAT3 protein expression demonstrated a significant increase in the sepsis group relative to the healthy group, with a significant difference also observed between the TCZ1, TCZ8, and TCZ10 groups and the healthy group. Analysis of pSTAT3 levels indicated a significant difference in the sepsis group compared to the healthy group. No significant difference was found between the TCZ1, TCZ6, TCZ8, and TCZ10 groups and the healthy group, while the TCZ16 group was statistically significantly different than the healthy group. Examination of SOCS3 protein expression revealed no significant difference between the sepsis group and the healthy group, whereas a significant increase was noted in the TCZ6 and TCZ16 groups compared to the healthy group (Fig. 5) (Table 2).

**Table 2:** The JAK2/STAT3/SOCS3 expression in kidney tissues of TCZ treatment groups was measured by immunohistochemistry

Groups	JAK2	pJAK2	STAT3	pSTAT3	SOCS3
Healthy	0,83 ± 0,40^aA^	0,16 ± 0,40^aB^	0,83 ± 0,40^aA^	0,16 ± 0,40^aB^	0,00 ± 0,00^aB^
Sepsis	3,00 ± 0,00^bA^	0,83 ± 0,40^bB^	2,83 ± 0,40^bA^	1,83 ± 0,40^bC^	1,16 ± 0,40^bB^
TCZ1	0,83 ± 0,40^cA^	0,16 ± 0,40^aB^	0,83 ± 0,40^aA^	0,00 ± 0,00^aB^	0,16 ± 0,40^aB^
TCZ6	2,83 ± 0,40^bA^	0,33 ± 0,51^aB^	2,66 ± 0,51^bA^	0,83 ± 0,40^cC^	1,83 ± 0,40^cD^
TCZ8	3,00 ± 0,00^bA^	0,33 ± 0,51^aB^	1,83 ± 0,40^cC^	0,16 ± 0,40^aB^	0,83 ± 0,40^bD^
TCZ10	1,66 ± 0,51^dA^	0,16 ± 0,40^aB^	1,66 ± 0,51^aA^	0,00 ± 0,00^aB^	0,00 ± 0,00^aB^
TCZ16	3,00 ± 0,40^bA^	0,83 ± 0,40^bB^	2,66 ± 0,51^bA^	1,83 ± 0,40^bC^	1,83 ± 0,40^cC^

a, b, c, d denote differences between groups in the same column (p < 0.05). A, B, C, D denote differences between groups in the same row (p < 0.05)

### Timing differences in TCZ treatment reveal differences in survival rate

**Fig. 6:**
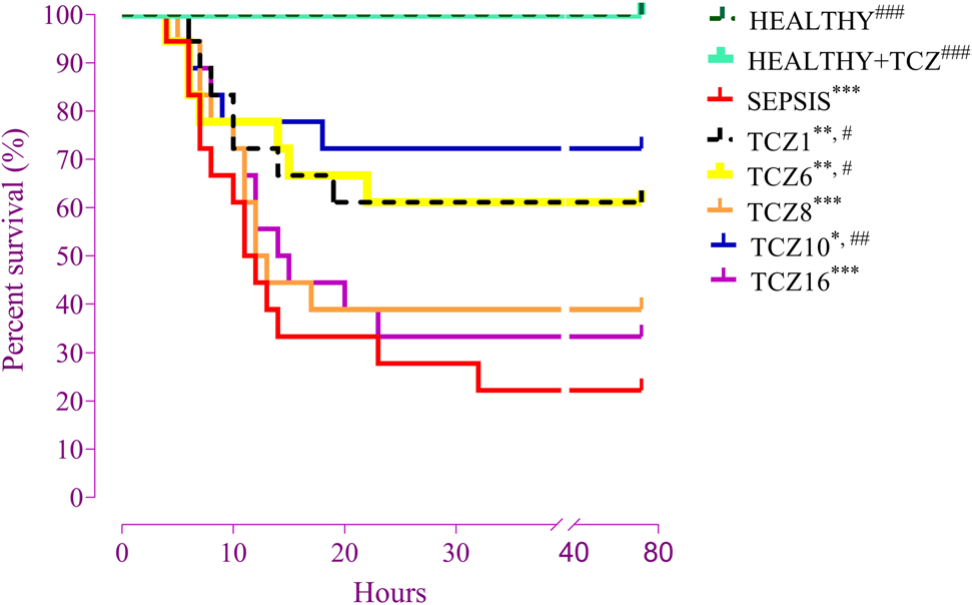
Kaplan–Meier survival analysis. *P < 0.05, **P < 0.01 and ***P < 0.001 vs. healthy group. #P < 0.05, ##P < 0.01 and ###P < 0.001 vs. sepsis group

Examining the survival analysis results, statistically significant differences were observed between the groups. The healthy and healthy + TCZ groups achieved a survival rate of 100% after 72 h of follow-up. In contrast, the sepsis group exhibited a statistically significantly lower survival rate than the healthy group and had the lowest survival rate among all groups. In all treatment groups, the TCZ10 group showed the highest survival rate. Specifically, the TCZ10 group demonstrated a statistically significantly higher survival rate compared to the sepsis group. For the TCZ1 and TCZ6 groups, which had identical survival rates, the survival rate was statistically significantly lower than the healthy group, although it remained statistically higher than the sepsis group. Examination of the TCZ8 and TCZ16 groups revealed a significantly decreased survival rate, closely approximating the sepsis group. The survival rates of the TCZ8 and TCZ16 groups were statistically significantly lower than those of the healthy group, with no statistically significant difference observed between these groups and the sepsis group (Fig. 6).

## Discussion

This study investigated the therapeutic potential of optimized treatment timing in sepsis immunotherapy, with TCZ treatment administered at varying intervals, using IL-6 as a biomarker in LPS-induced sepsis rats. It was necessary to examine serum IL-6 levels at narrow time intervals during the sepsis process and investigate the changes in the outcomes of systemic inflammation caused by IL-6 on organ injury over time to understand IL-6’s behavior in sepsis. For this reason, in the initial phase of our study, alterations in 24-h serum IL-6 levels, lung injury, and kidney injury, as well as their interrelationships, were assessed in rats with LPS-induced sepsis. The data from our initial study indicate that IL-6 exhibited an upward trend in the early stages of sepsis and reached a peak during this initial phase, followed by a decline to a low and stable level. Our findings align with those form a study investigating the clinical significance of the cytokine network in sepsis. In that study, it was reported that IL-6 exhibited the most significant increase in the early period compared to other cytokines examined, specifically IL-1β, IL-8, TNF-α, and Monocyte Chemoattractant Protein-1 (MCP-1) (Matsumoto et al. 2018). These results indicate that IL-6 is a potential biomarker for identifying the early proinflammatory phase in sepsis and distinguishing the subsequent immunosuppressive period. IL-6 is a potential prognostic and diagnostic marker for sepsis, and it is necessary to evaluate its levels in patients with sepsis or suspected sepsis (Song et al. 2019a). Our study examines the time-dependent changes in IL-6 in sepsis at small intervals. It provides valuable additional information to the literature regarding IL-6’s behavior in sepsis.

The lung is highly susceptible to damage during sepsis, with excessive inflammation, acute respiratory distress syndrome (ARDS), or acute lung injury (ALI) prevalent in patients with sepsis; it is the most frequent cause of ARDS/ALI (Sessler and Bloomfield 1996). For these reasons, monitoring the progression of lung injury in sepsis at short time intervals is essential. Our findings indicate that lung injury commenced after IL-6 levels were elevated. In contrast to the initial peak observed in IL-6 levels, the apex of lung injury manifested at a later and was corroborated by the negative result in the correlation test. Cytokines play a fundamental role in developing ARDS/ALI in sepsis. Cytokines induce multiple intracellular signaling pathways and coordinate the recruitment of various immune system components to the lung tissue (Kim et al. 2021; Lee and Choi 2021; Li et al. 2020). Lung injury in sepsis results from inflammation and a cytokine storm induced by immune system elements within the lung tissue (Li et al. 2022). Other findings show that tissue damage ensues following excessive cytokine release in the systemic circulation and tissue. Our study results corroborate this occurrence. Sepsis-induced kidney injury is characterized by acute dysfunction and organ injury, associated with long-term adverse outcomes correlated with the severity of the initial injury. This condition has been linked to an elevated risk of in-hospital mortality (Bellomo et al. 2017). Our data show that kidney injury began to manifest with the breakdown of the IL-6 peak, and that the peak of kidney injury occurred significantly later than the IL-6 peak. The significant negative correlation between IL-6 levels and kidney injury confirmed this. In addition, our study demonstrated that sepsis-induced lung injury developed earlier than kidney injury.

Examining the relationship between serum levels in IL-6, lung injury, and kidney injury was crucial in predicting the timing of treatment application in sepsis patients. Frequent monitoring of IL-6 serum levels can provide critical insights into the patient’s immune state, the potential direction of immune state changes, the extent and progression of lung injury, the onset timing of kidney injury, whether peak injury levels have been reached, and the estimated time of onset of sepsis. No specific FDA-approved therapeutic agents for treating sepsis are described in the literature (Doganyigit et al. 2022). For decades, clinical trials aiming to mitigate excessive inflammation in sepsis have been unsuccessful, leading to the characterization of sepsis as a pharmaceutical graveyard (Marques et al. 2023b). However, anti-inflammatory treatments such as cytokine blockers, have been remarkably successful in treating COVID-19, also known as viral sepsis, given its sepsis-like pathogenesis (Marques et al. 2023a). Thus, it has become evident that lessons must be derived from COVID-19 for the treatment of sepsis (Remy et al. 2020). In sepsis cases characterized by immune imbalance, it is crucial to safeguard the patient from both excessive inflammation and hypoinflammation (Marques et al. 2023a). Evaluating the literature, it is clear that the timing of an immunosuppressive intervention in sepsis is vital. To successfully employ anti-cytokine drugs like TCZ, which is, commonly used in COVID-19, treatment should focus on the timing and, consequently, the patient’s immune status in treating sepsis (Li et al. 2023).

We designed our second study to substantiate the hypothesis that optimal TCZ treatment administered in varying immune states would result in distinct outcomes in sepsis. Molecular and histopathological examinations on lung and kidney tissue following TCZ treatment indicated that the timing of TCZ administration impacts proinflammatory cytokine levels and histopathology differently. Cytokines are protein regulators that manage the immune response to infection and inflammation. Proinflammatory cytokines function as endogenous pyrogens, enhancing the synthesis of secondary mediators and other proinflammatory cytokines, while stimulating the production of acute phase proteins or attracting inflammatory cells (Chaudhry et al. 2013). Proinflammatory cytokine levels are closely associated with sepsis pathogenesis. Suppression of cytokine production or cytokine blockade may reduce the elevated proinflammatory cytokine load in sepsis and yield positive outcomes in patients. A decreasing trend in IL-6 values in patients during sepsis follow-up is thought to be associated with a better prognosis in sepsis (Kumar et al. 2009). Corticosteroids have been shown to suppress cytokine production and reduce mortality in sepsis cases when administered at low doses and for extended durations (Chaudhry et al. 2013). A prior study showed significant reductions in plasma IL-6 and TNF-α levels in rats following sepsis induction with Androstenediol administration. In addition, the survival rate improved in septic rats after treatment with Androstenediol (Suzuki et al. 2006). These findings indicate that reducing proinflammatory cytokine levels in sepsis is a therapeutic target. While reviewing our study results, we found that significant therapeutic successes were achieved with TCZ treatment in specific hour groups. Notably, the results for the 1st hour and 10th hour groups were closer to those of the healthy group, with proinflammatory cytokine levels markedly lower than in the sepsis group. When examining the proinflammatory cytokine levels and histopathology scores in the lung and kidney tissue of the TCZ1 group, treated in the 1st hour, the low levels observed indicated the effects of early immunosuppressive treatment on sepsis-related inflammation. Studies on the timing of TCZ application in COVID-19 showed that early TCZ treatment reduces C-reactive protein levels more effectively than standard treatment. Researchers advocate for early TCZ application, anticipating that it modulates the cytokine storm and prevents lung injury (Duran-Mendez et al. 2021; De Rossi et al. 2020). Contrary to expectations that results would worsen chronologically from an early-hour treatment to a later-hour treatment due to the early cytokine blockade mitigating the initial excessive inflammatory burden of sepsis, the outcomes at the 10th hour were unexpectedly superior. Treatment at the 10th hour yielded better results than those performed at the 6th and 8th hours. A study evaluating the efficacy of TCZ treatment in COVID-19 based on initial IL-6 levels may elucidate this discrepancy. Examination of the study’s results indicated that patients with initial IL-6 levels ≥ 100 pg/ml in the TCZ treatment group had worse clinical outcomes compared to patients with initial IL-6 levels < 100 pg/ml (Li et al. 2021). Given that elevated IL-6 levels indicate a significant inflammatory burden in sepsis, the study demonstrated that TCZ application during the cytokine storm period, characterized by hyperinflammation, is less effective. This may partially account for the greater efficacy of TCZ treatment at the 10th hour compared to treatments administered at the 6th and 8th hours. These findings indicate that the timing of TCZ application in sepsis can yield varying effects on tissue proinflammatory cytokine levels.

In addition to examining the levels of proinflammatory cytokines in the tissue, it was necessary to investigate the signaling pathways that trigger inflammation. This approach would enable understanding how inflammation progresses at the molecular level in the tissue, beyond the immediately measured cytokine levels. One of the principal signaling pathways of inflammation that predominates in sepsis is the JAK/STAT signaling pathway (Clere-Jehl et al. 2020). The JAK/STAT signaling pathway is responsible for cytokine production, immune cell recruitment and activation, and the initiation of adaptive responses. It plays a crucial role in the inflammatory response mediated by cellular immune factors (Huang et al. 2016). In sepsis, the JAK2/STAT3 pathway is specifically induced by IL-6, thereby initiating the proinflammatory effects of this cytokine through the generation of IL-6 trans-signaling (Zanders et al. 2022). SOCS3 is a negative feedback inhibitor of cytokine signaling by inhibiting JAK1 and JAK2 (Babon et al. 2012). Upon examination of our results, the expression and phosphorylation of the JAK2/STAT3 signaling pathway were significantly elevated in groups with a heavy inflammatory load, such as the sepsis and TCZ16 groups. They were at lower levels in groups with a lower inflammatory load, such as the TCZ1 and TCZ10. In addition, SOCS3, a suppressor of the JAK2/STAT3 pathway, exhibited higher expression in the sepsis and TCZ16 groups, as anticipated, in response to high inflammation. Analysis of kidney tissue revealed that the JAK2/STAT3 signaling pathway exhibited high expression in the sepsis and TCZ16 groups. The elevated JAK2/STAT3 expression values in the TCZ16 group, the late-stage treatment group, indicated the limitations of late-stage TCZ treatment in mitigating inflammation. In tissue proinflammatory cytokine measurements, the TCZ1 and TCZ10 groups, which exhibited lower cytokine levels than the other groups, also attenuated IL-6-induced JAK2/STAT3 signaling. In addition, as demonstrated in the initial study, it was found that JAK2/STAT3 levels were lower in the kidney tissue than in the lung tissue, attributed to the peak level of kidney injury occurring later. In a study conducted on COVID-19, it was reported that Ruxolitinib exhibited therapeutic effects by inhibiting the JAK/STAT pathway in the treatment of pulmonary inflammation characterized by cytokine release syndrome (La Rosée et al. 2020). Concurrently, in a sepsis study, Pectolinarigenin mitigate sepsis-induced kidney injury by inhibiting the JAK2/STAT3 pathway (Tan et al. 2023). Thus, effective suppression of the JAK2/STAT3 pathway appears to have substantial therapeutic value in sepsis and diseases with comparable pathogenesis. The results of the TCZ1 and TCZ10 groups in our study elucidate how the timing of treatment can yield distinct outcomes in the molecular progression of inflammation.

The results of the survival study indicate that the TCZ10 group most closely approximated the healthy group. Although the TCZ1 group exhibited lower proinflammatory cytokine levels, histopathological scores, and JAK/STAT expression, survival outcomes lagged behind those of the TCZ10 group. Unexpectedly, the TCZ treatment administered at the 10th hour yielded a result outside the chronological treatment order. These findings suggest that early immunosuppressive treatment may impact mortality in late-stage sepsis. Contrary to prior assumptions, it has already been established that interventions targeting excessive inflammation in the early stages of sepsis do not improve mortality (Liu et al. 2022). In the early stages of sepsis, the immune system overcomes the pathogen and immune balance is swiftly restored; however, if the immune system fails to compete with the pathogen, immune imbalance occurs, and patients may experience persistent inflammation, immunosuppression, and catabolism syndrome, and be susceptible to secondary infections and endure long-term immunosuppression (Pandharipande et al. 2013; Liu et al. 2022). While some researchers advocate for early TCZ treatment in diseases such as COVID-19, the comprehensive analysis in our study indicates that early immunosuppressive treatment is not the optimal choice when considering longer-term outcomes such as survival studies. This conclusion holds even if inflammation-related parameters and signaling pathway activation are reduced at the end of the 24th hour, as observed in the TCZ1 group with early immunosuppressive treatment in sepsis (Duran-Mendez et al. 2021; De Rossi et al. 2020). Therefore, even if the clinical and laboratory findings of patients undergoing early immunosuppression are favorable for a certain period, their long-term outcomes should be monitored meticulously. In addition, to prevent early organ damage due to excessive inflammation, immunomodulatory treatment options should not be disregarded in protecting patients receiving early immunosuppressive treatment for long-term immunosuppression.

In conclusion, this study aimed to support the selection of treatment timing by determining the relationship between the relevant biomarker and sepsis-induced organ damage in biomarker-guided sepsis immunotherapy and demonstrate that immunosuppressive treatment applied in sepsis at different timings can yield unexpected results according to the reactions of the patient’s immune phenotype rather than early or late treatment application.

## Data Availability

The research data will be made available upon reasonable request.
