# Correction: The impact of tocilizumab treatment on the severity of inflammation and survival rates in sepsis is significantly influence by the timing of administration

**DOI:** 10.1007/s10787-025-01748-4

**Published:** 2025-04-26

**Authors:** Taha Tavaci, Zekai Halici, Elif Cadirci, Mustafa Ozkaraca, Kamber Kasali

**Affiliations:** Sakarya University Faculty of Medicine, Sakarya, Türkiye

**Correction: Inflammopharmacology (2025) 33:1393–1405** 10.1007/s10787-025-01649-6

In this article, the open access funding information was incorrectly published in the original publication. The incorrect and correct versions are given below.

Incorrect version:

Open access funding provided by the Scientific and Technological Research Council of Türkiye (TÜBİTAK

Correct version:

This study supported by the Ataturk University Scientific Research Projects Coordination Unit (grant number: TDK-2021-9459). The funding agency had no role in study design, collection and analyses of data, decision to publish, or preparation of the article.

The original article has been corrected.

